# Development of intron polymorphism markers in *major latex*-*like protein* gene for locality-level and cultivar identification of *Salvia miltiorrhiza*

**DOI:** 10.1186/s40064-016-3611-5

**Published:** 2016-11-04

**Authors:** Hongtao Wang, Na Hao, Lei Chen, Guisheng Li

**Affiliations:** 1 School of Life Sciences, Yantai University, Yantai, People’s Republic of China; 2 School of Pharmacy, College of Pharmacy, Yantai University, Yantai, People’s Republic of China

**Keywords:** *Salvia miltiorrhiza*, Locality-level identification, Cultivar identification, Major latex-like protein, Intron polymorphism

## Abstract

**Background:**

*Salvia miltiorrhiza* (Danshen) is one of the most widely used medicinal herbs in traditional Chinese medicine. Locality-level and cultivar identification is of great importance not only for protecting highest therapeutic effectiveness of Daodi Danshen, but also for the genetic conservation and utilization of existing *S. miltiorrhiza* populations.

**Results:**

Intron polymorphisms including SNPs (single nucleotide polymorphisms) and indels were exploited in *major latex*-*like protein* (*MLP*) gene. Based on these markers, genetic relationships among *S. miltiorrhiza* cultivar and populations in different locations were evaluated by constructing a dendrogram. Moreover, *S. miltiorrhiza* specimens from Laiwu region were geographically distinguishable by the developed SNP marker. A 204 bp-indel marker was exploited for the first space breeding cultivar Luyuan Danshen-1 (LD-1), and an effective real-time PCR assay was successfully developed for fast screening of LD-1 among local landraces.

**Conclusions:**

*MLP* intron is a valuable DNA barcode for intra-specific study of *S. miltiorrhiza* populations, and the developed markers can serve as a useful tool for molecular identification of LD-1 cultivar and geographically distinct populations of *S. miltiorrhiza*.

**Electronic supplementary material:**

The online version of this article (doi:10.1186/s40064-016-3611-5) contains supplementary material, which is available to authorized users.

## Background

*Salvia miltiorrhiza* (Lamiaceae), known as Danshen in Chinese, is one of the most widely used medicinal herbs in traditional Chinese medicine. Its dried root or rhizome, commonly called ‘red sage’, has been used for centuries in the treatment of cardiovascular and cerebrovascular diseases (Zhou et al. 2005). *S. miltiorrhiza* contains two major groups of active compounds: the lipophilic diterpenoids and hydrophilic phenolic acids (Li et al. 2009). Modern pharmacological studies have demonstrated that these compounds are responsible for many therapeutic actions, such as anti-inflammatory, antibacterial, anti-carcinogenic, and antioxidant activities (Zhou et al. 2005; Hung et al. 2010; Wang 2010). Besides, *S. miltiorrhiza* has also become widely accepted as functional food due to its significant antioxidant capacity.

The Chinese term ‘Daodi’ refers to the medicinal material that is produced in specific geographic regions with designated natural conditions and ecological environment. The superior germplasm resource and suitable geographic regions are the key factors of the formation of Daodi medicinal material (Zhao et al. 2012). Shandong province is one of the most important Daodi production regions in China and has rich germplasm resources of *S. miltiorrhiza* (Song et al. 2010). Due to the decrease of wild resources, *S. miltiorrhiza* landraces of different production areas in Shandong province have become the major source of commercial Danshen herb. Nowadays, with the rapid development of space technologies, space breeding provides a new technical platform for screening new varieties of crops. Compared to the plant growing conditions on Earth, the effects of space environment, such as high-energy ion radiation, microgravity, space magnetic field, ultra vacuum, offer great opportunities of genetic mutagenesis. In 2004, the dried seeds of *S. miltiorrhiza* were carried into space by China’s 20th retrievable satellite. After 18 days’ spaceflight with retrievable satellite, the returned seeds were screened by ground-based observation and selection. Luyuan Danshen-1 (LD-1) is the first successfully selected space breeding cultivar of *S. miltiorrhiza*, which was registered in Shandong Provincial Department of Agriculture in 2013. LD-1 cultivar produces a higher content of salvianolic acid and yield than local landraces. Besides, *S. miltiorrhiza* from different geographic populations have been shown to contain different contents of active compounds (Ran et al. 2008). For example, *S. miltiorrhiza* from Laiwu region was regarded to be more potent than those grown in other regions. Therefore, locality and cultivar identification is very important not only for protecting the quality of Shandong Daodi Danshen, but also for genetic conservation and utilization of existing *S. miltiorrhiza* populations.

Traditional means for authentication of *S. miltiorrhiza* populations commonly rely on morphological characteristics. These approaches, however, are sometimes unreliable because most of *S. miltiorrhiza* populations and cultivar are morphologically similar (Zhang et al. 2013), which renders their differentiation subjective and error-prone. In recent years, various molecular markers have been developed for investigating the genetic diversity of *S. miltiorrhiza*, including RAPD (Guo et al. 2002), AFLP (Wang et al. 2007), CoRAP (Wang et al. 2009), ISSR (Zhang et al. 2013), and SRAP (Song et al. 2010). While previous studies focused on the population structure and rich genetic diversity within cultivated populations in different producing areas of China, no research attention has been paid to the cultivar and locality-level identification of *S. miltiorrhiza*. In this study, we aimed to develop intron polymorphism markers in *major latex*-*like protein* (*MLP*) gene to evaluate genetic relationships among *S. miltiorrhiza* populations in different locations, and constructed a simple method for cultivar and locality-level discrimination of *S. miltiorrhiza* in Shandong province.

## Results

### PCR of *MLP* intron and sequence analysis

**Fig. 1:**
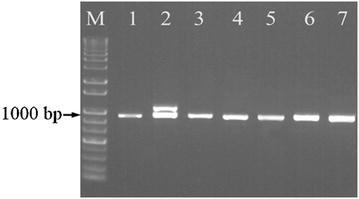
PCR products of MLP introns of *S. miltiorrhiza* samples from different regions. *Lane M* 1000 bp DNA ladder, *lane 1* Yantai, *lane 2* LD-1, *lane 3* Linyi, *lanes 4, 5,* Laiwu, *lanes 6, 7* Weifang

**Fig. 2:**
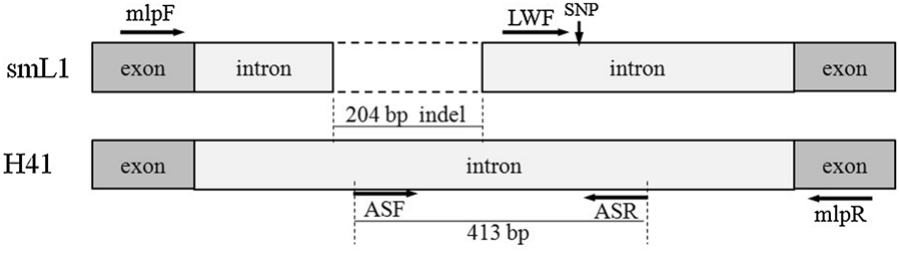
Graphical overview of the positions of the primer sets used in this study

In order to check the feasibility of the designed primers mlpF and mlpR, seven *S. miltiorrhiza* samples from four different regions were randomly chosen for amplification of *MLP* intron. Figure 1 showed that all the samples were successfully amplified with their single bands, except that the LD-1 cultivar generated two fragments. *MLP* introns of the 120 *S. miltiorrhiza* samples were amplified and sequenced, and a total of 10 genotypes were detected. As shown in Additional file 1: Fig. S1, two genotypes were discovered respectively in *S. miltiorrhiza* (smL1, smL2) and *S. miltiorrhiza* f. *alba* (smf1, smf2) in Laiwu, two genotypes were detected in Weifang population (smW1, smW2) and LD-1 cultivar (H41, H42), and samples in Yantai (smYt) and Linyi (smLy) showed their unique genotypes. The compiled DNA sequences were registered in GenBank with accession numbers of KU891682–KU891691. Multiple sequence alignment results showed that intron length polymorphisms and SNPs were exploited in *MLP* intron. As shown in Fig. 2 and Additional file 1: Fig. S1, a 204 bp-insertion was determined in the longer fragments (H41) of LD-1, compared with the shorter band (H42) and fragments of other landrace samples. Besides, at the 562 bp nucleotide position of Additional file 1: Fig. S1, a SNP unique to Laiwu population was discovered. Samples from Laiwu region contain nucleotide T, which was replaced by C at the same position in the samples from other geographic regions.

### Genetic relationship among different populations and specific primer design

**Fig. 3:**
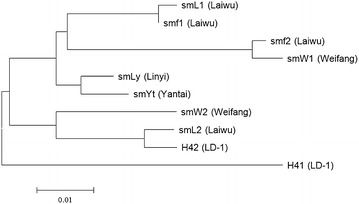
Dendrogram constructed on the basis of *MLP* intron sequences using the neighbor-joining method

The polymorphic *MLP* intron sequences including SNPs and indels of different *S. miltiorrhiza* populations and cultivar were used to construct a neighbor-joining dendrogram. As shown in Fig. 3, the LD-1 cultivar (H41 type) formed a separate clade showing less similarity with the other populations, indicating that the space-induced mutagenesis was indeed occurred in LD-1. The sister large group was divided into two subclusters: one consisted of samples of Weifang (SMW2 type), Laiwu (SML2 type), and LD-1 cultivar (H42 type), and the other branch contained specimens from Laiwu (SML1 type), Weifang (SMW2 type), Linyi, and Yantai. These results demonstrated that the landrace from which LD-1 cultivar originated has a close relationship with the samples in Laiwu (smL2), and there were gene exchange between populations of Laiwu and Weifang.

To authenticate *S. miltiorrhiza* populations originated from Laiwu and the LD-1 cultivar, specific primers were designed based on their unique sequences. Primer LWF (5′-CATACCCACTATACATTCTTGAT-3′) was designed for specific identification of *S. miltiorrhiza* from Laiwu, by substituting of G for A at the third base from 3′ end. Primers ASF (5′-CTTTACTTTCGGCACTGGTT-3′) and ASR (5′-ATCCGTCTCACTTATCTTGG-3′) were designed for specific authentication of LD-1from other local landraces. The relative positions of these primers were shown in Fig. 2 and Additional file 1: Fig. S1.

### Molecular authentication of Laiwu population and LD-1

**Fig. 4:**
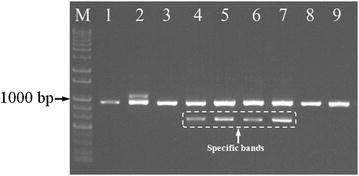
Multiplex PCR products using primers mlpF, mlpR, and LWF. *Lane M* 1000 bp DNA ladder, *lane 1* Yantai, *lane 2* LD-1, *lane 3* Linyi, *lanes 4–7* Laiwu, *lanes 8*, *9* Weifang

**Fig. 5:**
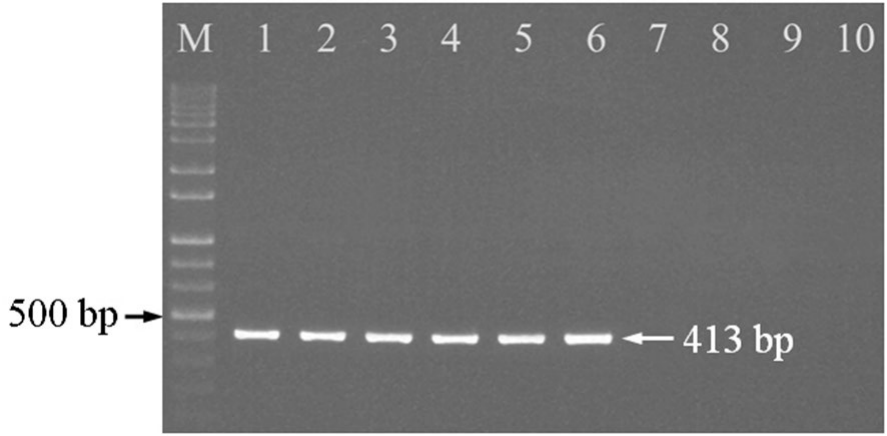
PCR products using primers ASF and ASR. *Lane M* 1000 bp DNA ladder, *lanes 1–6* LD-1, *lane 7* Yantai, *lane 8* Linyi, *lane 9* Laiwu, *lane 10* Weifang

**Fig. 6:**
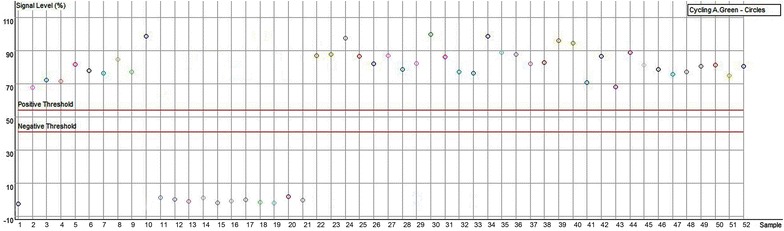
Endpoint analysis for discriminating of LD-1 from other samples. *1* No template control, *21* negative control, *52* positive control, *11–14* Laiwu, *15–16* Weifang, *17–18* Linyi, *19–20* Yantai, *2–10* 22–51: LD-1

Molecular authentication of *S. miltiorrhiza* from Laiwu population was performed using multiplex PCR. As shown in Fig. 4, all the samples generated the same fragments as shown in Fig. 1, but only those from Laiwu produced their specific bands representing the T allele. Similarly, primers ASF and ASR amplified LD-1-specific band of 413 bp, but no PCR products were detected in other landraces (Fig. 5). In order to check the accuracy of the indel marker and construct a fast assay of LD-1, real time PCR was conducted and endpoint analysis method was used for identification of LD-1. Figure 6 showed that 40 LD-1 samples were easily discriminated from other landraces by their signal levels compared with the positive threshold. Therefore, LD-1 cultivar and *S. miltiorrhiza* from Laiwu population can be successfully authenticated by the indel and SNP markers in *MLP* intron.

## Discussion

Locality-level and cultivar identification is of great importance to ensure highest therapeutic effectiveness and help select the best strains of *S. miltiorrhiza.* There have been a number of studies developing molecular markers for *S. miltiorrhiza* grown in different geographical regions (Wang et al. 2007; Song et al. 2010; Zhang et al. 2013). Although high genetic diversity was revealed among *S. miltiorrhiza* populations, effective locality-level identification is difficult because molecular markers such as RAPD, ISSR, SSR, CoRAP, and SRAP require more primer pairs to obtain enough PCR products and are easily affected by PCR conditions. On the other hand, commonly used DNA barcodes like ITS2 and psbA-trnH are not polymorphically informative among *S. miltiorrhiza* populations to develop SNP markers (Chen et al. 2010). In this study, intron polymorphism (SNP and indel) markers were exploited in *MLP* gene and proved to be effective for molecular identification of LD-1 cultivar and laiwu population of *S. miltiorrhiza*.

A host of SNPs and indels existed in *MLP* introns among and within different populations. The high genetic polymorphism among populations of different regions may greatly attribute to the influence of geographic environments. The high level of genetic polymorphism within populations (such as Laiwu and Weifang) may result from the traditional practice of mixed cultivation of seeds without deliberate selection. Especially in Laiwu region, *S. miltiorrhiza* var. *miltiorrhiza* and *S. miltiorrhiza* f. *alba* are often planted together in one field, where cross-pollination occurs at a high rate. It is worthy to note that a 204 bp-insertion was detected in the intron of *MLP* gene in cultivar LD-1, which indicated that space mutagenesis breeding is an efficient way for creating new germplasms of *S. miltiorrhiza*. The association between the indel marker and the agronomic characters of LD-1 needs further study.

Molecular authentication of *S. miltiorrhiza* from Laiwu was achieved by exploiting a SNP site unique to Laiwu population. *S. miltiorrhiza* samples from Laiwu can be easily discriminated from other populations by the specific amplicon representing T allele in *MLP* intron. The established multiplex PCR was conducted many times with 20 specimens and showed 100% accuracy. Intron length is relatively stable because indels of nucleotides do not tend to occur as often as SNPs (Ching et al. 2002). An indel marker was developed for the space breeding cultivar LD-1, and the real time PCR with endpoint analysis enabled clear identification and fast screening of LD-1 from landraces of different populations. Therefore, a simple and convenient method for identification of *S. miltiorrhiza* populations and cultivar was developed based on the intron polymorphism markers in *MLP* gene.

## Conclusions

In this study, we demonstrated that the intron of *MLP* gene is a valuable DNA barcode for intra-specific polymorphism exploitation of *S. miltiorrhiza*. The markers developed in this study can serve as a useful tool for marker-assisted selection of LD-1 cultivar and geographically distinct populations of *S. miltiorrhiza*, and the method may also be applied to closely related populations and cultivars of other medicinal plants.

## Methods

### Plant materials and DNA isolation

A total of 120 samples of *S. miltiorrhiza* were collected from four geographically distinct populations in Shandong province. All the specimens were morphologically identified by Prof. Guisheng Li and deposited in Star Aviation Breeding Company (Yantai, Shandong). According to their localities and variety, the plant samples were divided into six groups for analysis (Table 1). Genomic DNA of the 120 individuals were respectively isolated by using a Plant DNA isolation kit (Easypure Plant Genomic DNA Kit, TransGen Biotech), according to the manufacturer’s instructions. The prepared 120 DNA samples were stored at −20 °C for further analysis.

**Table 1:** Plant samples used in this study

Variety/cultivar	Location	No. of samples	Voucher specimen
*S. miltiorrhiza* var. *miltiorrhiza*	Yantai	15	SMyt01
*S. miltiorrhiza* f. *alba*	Laiwu	15	SMflw11, SMflw17
*S. miltiorrhiza* var. *miltiorrhiza*	Laiwu	15	SMlw23, SMlw26
*S. miltiorrhiza* var. *miltiorrhiza*	Linyi	15	SMly31
*S. miltiorrhiza* var. *miltiorrhiza*	Weifang	20	SMwf42, SMwf47
Luyuan Danshen-1	Yantai	40	H401

### PCR of *MLP* intron and sequence analysis

PCR amplification of *MLP* intron were conducted using primers mlpF (5′-TTTAGGCACAAACCACATGATC-3′) and mlpR (5′-CGTGGGCTGTAATAACGAATG-3′), which were designed according to the exon sequences flanking the target intron (GenBank accession GQ923782). The 20 μL reaction mixture consist of 10–50 ng of template DNA, 0.5 μM of each primer, and 10 μL of 2× EasyTaq PCR SuperMix (TransGen Biotech). The mixtures were heated at 94 °C for 4 min followed by 33 cycles of 30 s at 94 °C, 30 s at 60 °C, and a 1-min extension at 72 °C with final extension reaction at 72 °C for 7 min. PCR products were analyzed via 1.0% agarose gel electrophoresis and visualized by ethidium bromide staining under UV.

The PCR products were cut and recycled with an EasyPure Quick Gel Extraction kit (TransGen Biotech), according to the manufacturer’s instructions. The purified products were ligated into the pGEM-T Easy vector (Promega, USA) and transformed into competent *E. coli* DH5α cells. After transformant selection, white clones were cultivated in LB liquid medium at 37 °C overnight with shaking. Plasmid DNA was isolated with a Plasmid DNA MiniPrep kit (TransGen Biotech) and sequenced for both forward and reverse directions on an automatic DNA sequencer (ABI PRISM 3700, USA). DNA sequences were assembled using SeqMan software, and multiple sequence alignments were conducted using the Clustal Omega program (Sievers and Higgins 2014).

### Phylogenetic analysis and specific primer design

Phylogenetic analysis was performed to construct a dendrogram using the neighbor-joining module of the MEGA software (Tamura et al. 2011). Based on the SNP site representing Laiwu population, primer was designed by introducing an additional mismatch for specific identification of *S. miltiorrhiza* from Laiwu population (Drenkard et al. 2000). Primers specific to LD-1 were designed according to the insertion sequence compared to the other populations.

### Molecular authentication of Laiwu population and LD-1

Molecular authentication of Laiwu population was conducted with primers mlpF, mlpR, and LWF. Primers mlpF and mlpR were included in all reactions and served to provide a control PCR product. PCR was performed in a total volume of 20 μL, and the reaction mix consisted of each of the primers at a concentration of 0.5 μM, 10–50 ng of template DNA, and 10 μL of 2× EasyTaq PCR SuperMix (TransGen Biotech). The PCR amplification profile was identical with described above. Molecular identification of LD-1 cultivar was performed with primers ACF and ACR. The 20 μL reaction mixture consist of 10–50 ng of template DNA, 0.5 μM of each primer, and 10 μL of 2× EasyTaq PCR SuperMix (TransGen Biotech). The mixtures were heated at 94 °C for 4 min followed by 33 cycles of 30 s at 94 °C, 30 s at 62 °C, and a 1-min extension at 72 °C with final extension reaction at 72 °C for 7 min. To validate the accuracy of the indel marker and construct a fast assay of LD-1, a real-time PCR was conducted. The 10 μL reaction mixture consisted of 5–50 ng DNA, 5 μM of each primer, and 5 μL 2× SYBR Green I Mastermix (SensiMixPlus SYBR, Australia). The PCR cycling profile was as follows: 10 min of activation at 95 °C, followed by 40 cycles of a three-step thermal profile involving 10 s at 95 °C for denaturation, 15 s at 62 °C for combined annealing, and 20 s at 72 °C for extension. The melting analysis condition was performed with a ramp from 85 to 98 °C, rising by 1 °C at each step. Endpoint analysis method was used for the determination of LD-1.

## Additional file

**Additional file 1: Fig. S1.** Comparison of *MLP* intron sequences of different populations and LD-1 cultivar.

## Abbreviations

SNP: single nucleotide polymorphism
*MLP*: *major latex*-*like protein*
LD-1: Luyuan Danshen-1
RAPD: random amplified polymorphic DNA
AFLP: amplified fragments length polymorphism
CoRAP: conserved region amplification polymorphism
ISSR: inter-simple sequence repeat
SRAP: sequence related amplified polymorphism
SSR: simple sequence repeat
ITS: internal transcribed spacer
