# Supplementary figures and images for: Development of intron polymorphism markers in major latex-like protein gene for locality-level and cultivar identification of Salvia miltiorrhiza
Source: Springerplus. 2016 Nov 4;5(1):1919. doi: 10.1186/s40064-016-3611-5 (PMC5097057; doi:10.1186/s40064-016-3611-5)

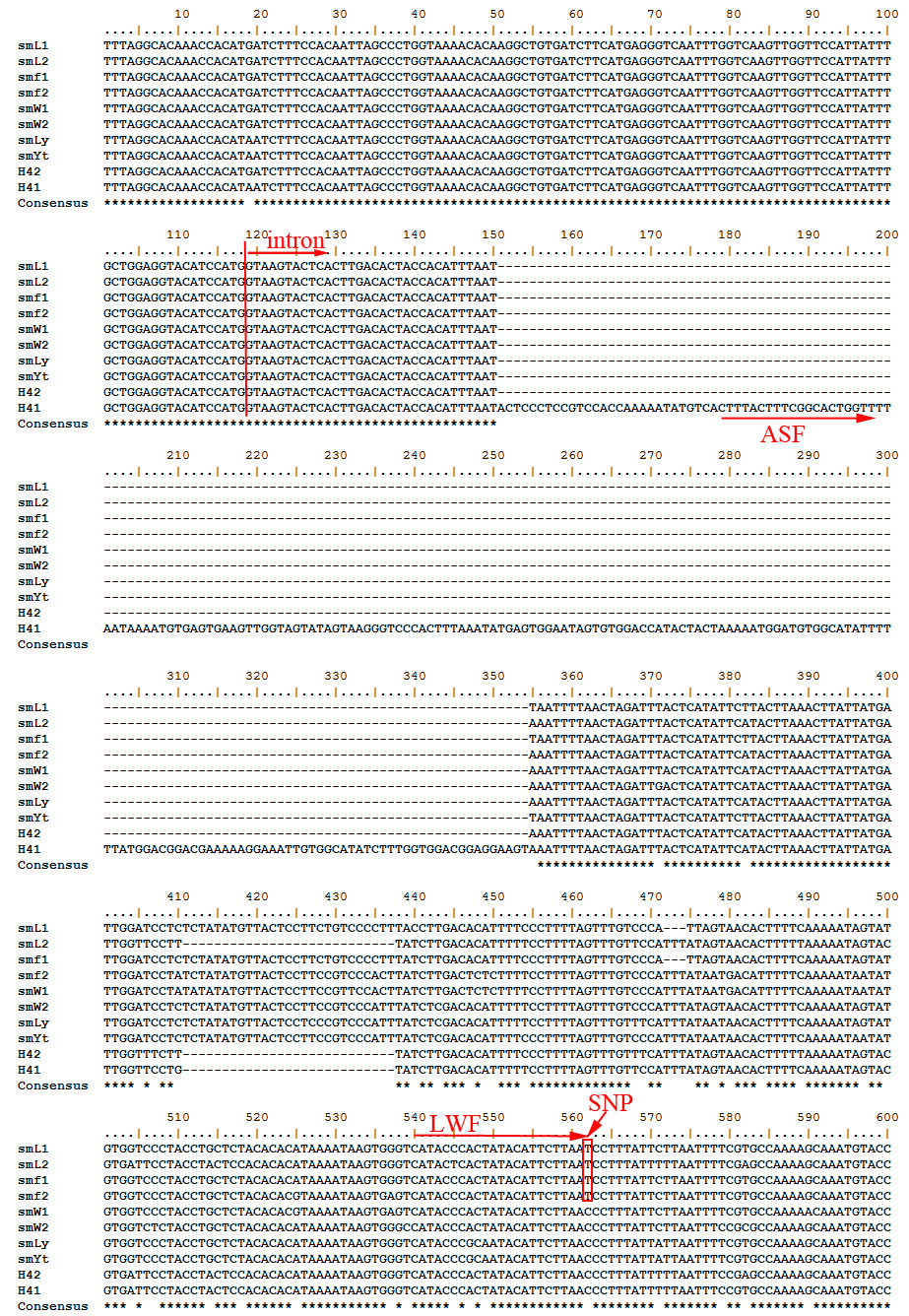

Supplement: Supplementary file 1 — Additional file 1: Fig. S1. Comparison of MLP intron sequences of different populations and LD-1 cultivar. [file 40064_2016_3611_MOESM1_ESM.zip › 40064_2016_3611_MOESM1_ESM/Supplementary Fig. 1-1.jpg]

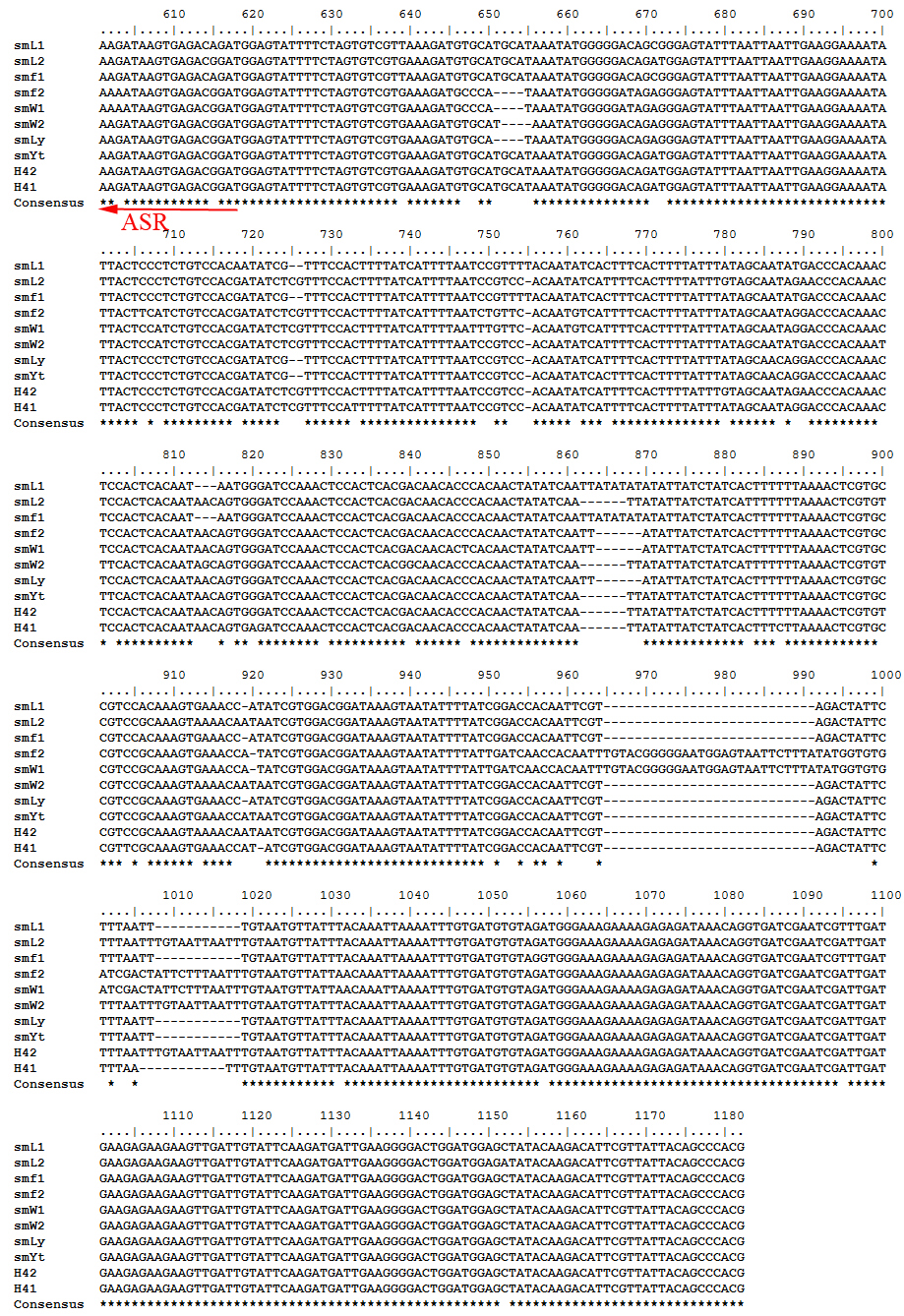

Supplement: Supplementary file 1 — Additional file 1: Fig. S1. Comparison of MLP intron sequences of different populations and LD-1 cultivar. [file 40064_2016_3611_MOESM1_ESM.zip › 40064_2016_3611_MOESM1_ESM/Supplementary Fig. 1-2.jpg]

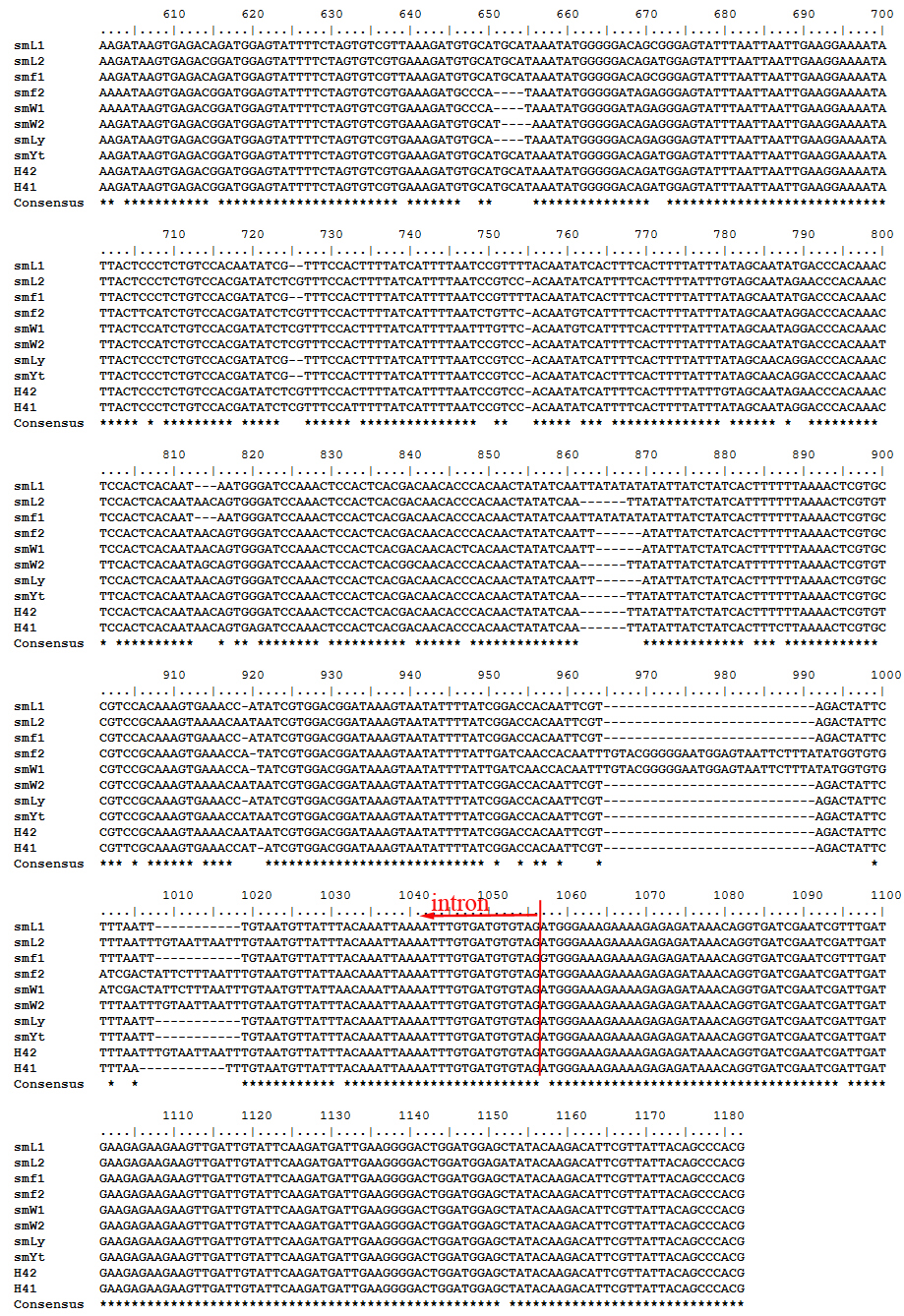

Supplement: Supplementary file 1 — Additional file 1: Fig. S1. Comparison of MLP intron sequences of different populations and LD-1 cultivar. [file 40064_2016_3611_MOESM1_ESM.zip › 40064_2016_3611_MOESM1_ESM/Supplementary Fig. 1-3.jpg]
